# High-Temperature Reactive Wetting of Natural Quartz by Liquid Magnesium

**DOI:** 10.3390/ma17061302

**Published:** 2024-03-11

**Authors:** Azam Rasouli, Artur Kudyba, Grzegorz Bruzda, Jafar Safarian, Gabriella Tranell

**Affiliations:** 1 Department of Materials Science and Engineering, Norwegian University of Science and Technology, 7491 Trondheim, Norway; jafar.safarian@ntnu.no (J.S.); gabriella.tranell@ntnu.no (G.T.); 2 Łukasiewicz Research Network–Kraków Institute of Technology, 30-418 Kraków, Poland; artur.kudyba@kit.lukasiewicz.gov.pl (A.K.); grzegorz.bruzda@kit.lukasiewicz.gov.pl (G.B.)

**Keywords:** natural quartz, reactive wetting system, high temperatures, product microstructure

## Abstract

High-temperature wetting of natural, high-purity quartz (SiO_2_) and liquid magnesium (Mg) was investigated at temperatures between 973 and 1273 K. Sessile drop experiments using the capillary purification (CP) procedure were carried out under an Ar gas atmosphere (N6.0), eliminating the native oxide layer on the surface of Mg melt. The results showed that the wetting behavior was strongly dependent on temperature. At 973 and 1073 K, the wetting system displayed relatively large contact angles of 90° and 65°, respectively, demonstrating modest wetting. The wetting increased to some extent by increasing the temperature to 1123 K with a wetting angle of 22°. However, the SiO_2_/Mg system demonstrated complete wetting at temperatures of 1173 K and above. Furthermore, interface microstructure examination showed different reaction product phases/microstructures, depending on the wetting experiment temperature.

## 1. Introduction

The metallothermic reduction of SiO_2_, including aluminothermic and magnesiothermic reduction, to produce Si for various applications has received increasing attention in recent years. These applications include high-purity Si and Si with special mesoporous structures for application, mainly in Li-ion batteries [1,2,3,4,5,6,7,8,9]. The difference in melting points of SiO_2_ as the oxide phase and metals like Mg and Al suggests that the metallothermic reduction reaction might occur between the solid oxide and the liquid metal, where the diffusion rate of metal and the wettability of SiO_2_ by the metal influence the reaction mechanism. While the latter has been thoroughly investigated, there are only a few studies on wettability in the SiO_2_/Mg system, particularly at higher temperatures [2,4,6,7,10,11,12].

Oxide–metal systems generally exhibit low wettability, where the wetting behavior might be improved through the formation of product compounds at the oxide–metal interface. Wetting systems, such as SiO_2_/Si, Al_2_O_3_/Si, MgO/Si, and Al/MgO, exhibit non-wetting behavior since chemical reactions between the metal and substrate only occur to a very limited extent. For example, the partial dissolution of oxygen in Si in the Si/SiO_2_ system and the formation of silicate layers (2MgO-SiO_2_ and 2MgO-2SiO_2_) in the MgO/Si system reportedly led to decreasing contact angles of a few degrees [13,14,15,16,17]. In contrast, reactive wetting systems, such as Mg/SiO_2_, Al/SiO_2_, Mg-Al/SiO_2_, and Mg/Al_2_O_3_, exhibit extensive chemical reaction with highly negative Gibbs energy, where the wettability is strongly affected by the chemical reaction at the metal–substrate interface. Marumo and Pask [18] studied the SiO_2_/Al system where the contact angle decreased with temperature as a result of the formation of various layers at the solid/liquid interface in the temperature range 933 to 1473 K (660 to 1200 °C). On the contrary, Shen et al. [19] reported a lack of wetting in the SiO_2_/Al system with a contact angle larger than 90° under an Ar-3% H_2_ atmosphere and 0.11 MPa pressure for temperatures between 1073 and 1523 K (800 and 1250 °C) where no product formation was detected at the SiO_2_/Al interface. In another work by Shi et al. [11], it was observed that the contact angle decreased when alloying Al with Mg in the SiO_2_/Al-Mg system. The contact angle changed from higher than 85° in the SiO_2_/Al system to less than 10° in the SiO_2_/Al-69%Mg system. The Mg/SiO_2_ system was studied by Shi et al. [12] over the temperature range 973 K to 1073 K using a sessile drop method under an Ar gas atmosphere. They found that there was good wetting of SiO_2_ by Mg, mainly due to the formation of Mg_2_Si in the reaction zone. In other words, the rate of spreading of a metal on an oxide substrate depends on the reaction rate, as well as the change in surface tension between liquid and vapor by, e.g., a dissolving product compound, a change in the viscosity of the liquid, and/or volume change due to product formation [11,12,18,19,20,21,22].

In our previous work on the kinetics of the reaction between Mg and SiO_2_, a noticeable difference between reaction kinetics at temperatures below 1173 K and higher temperatures was observed, which was primarily related to the diffusion rate of Mg. However, it was proposed that the reaction rate may be affected by the wetting behavior between different phases as well [23,24]. To the best of our knowledge, the SiO_2_/Mg system has not been studied at temperatures above 1073 K, as it can be challenging to conduct wetting experiments at such high temperatures due to the high vapor pressure of Mg and oxidation of the Mg source. Hence, a more in-depth understanding of the effect of temperature on wetting between reactants is beneficial to provide more detailed information about the mechanism of the Mg-SiO_2_ reaction. The aim of this work was to provide more detailed information on the wettability of high-purity natural quartz by liquid Mg, particularly at temperatures above 1173 K.

## 2. Materials and Methods

### 2.1. Materials

SiO_2_ disks (diameter of 16 mm and thickness of 3 mm) were prepared from high-purity natural quartz to be used as the substrate. The natural quartz contained 0.0106 wt% Al, 0.002 wt% Fe, 0.0023 wt% K, 0.001 wt% Na, 0.0009 wt% Mg, 0.0003 wt% Ca, 0.00042 wt% Ti, and 0.00003 wt% Mn as impurity elements measured by inductively coupled plasma–optical emission spectrometry (ICP-OES) [25]. The disks were ultrasonically cleaned in ethanol for 20 min before performing wetting tests. A commercial Mg with a purity of 99.98 wt% (supplied by Stanchem, Niemce, Poland) was used to make the liquid Mg metal drop. Mg pieces had a disk shape with dimensions adapted to the size of the graphite capillary making the drop (diameter of 10 mm and height of 15 mm). Before performing wetting tests, the Mg pieces were subjected to mechanical grinding with SiC papers to remove possible surface oxide layers, followed by ultrasonic cleaning in isopropanol for 5 min.

### 2.2. Procedure

**Figure 1:**
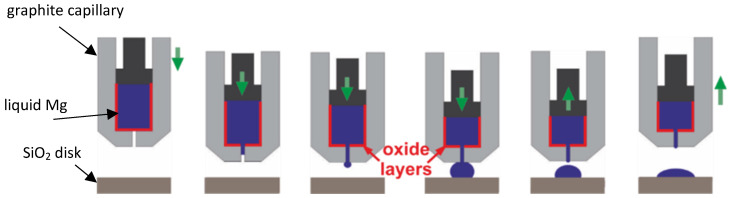
The scheme shows the separate heating of the tested couple of materials and the mechanical purification of a metal drop from the oxide film by squeezing a drop from the capillary in the capillary purification procedure [27].

High-temperature wettability tests of the SiO_2_/Mg system were performed by a classical sessile drop method using the capillary purification (CP) procedure. This procedure ensures non-contact heating of the materials couple under investigation (e.g., Mg and SiO_2_) combined with the in situ mechanical removal of the native oxide film from the Mg melt and deposition of the oxide-free Mg droplet on a substrate by squeezing liquid metal through a graphite capillary, as shown schematically in Figure 1. A more detailed description of the CP procedure is given elsewhere [26,27,28,29,30].

The de-greased and cleaned Mg pieces were directly put in a capillary located above the test substrate. Graphite capillaries were selected as “inert” containers to avoid alloy contamination by interactions between the molten Mg and the capillary. After positioning the samples inside the chamber, residual gases were evacuated using scroll and turbo-molecular pumps. When the total pressure inside the chamber reached approximately 5 × 10^−6^ mbar, the reactor was heated to the set temperature at a heating rate of 50 K/min. At 373 K, inert Ar gas (N6.0) was introduced into the chamber at a pressure of 760–850 mbar. The high-temperature wettability experiments of the SiO_2_/Mg system were carried out under an Ar gas flow at the following test temperatures: 973, 1073, 1123, 1173, 1223, and 1273 K. The test duration was 5 min at each temperature, after which the samples were cooled down to room temperature at a cooling rate of 50 K/min. During the wetting experiments, images of the SiO_2_/Mg couple were recorded in real time by a high-speed digital CCD camera (Microtron MC 1310, Microtron GmbH, Unterschleißheim, Germany) at 100 frames per second. A backlight was applied to enhance the contrast between the sessile drop couple profile and the background. The resulting images were analyzed using ASTRAView© software 2.0 developed by CNR-ICMATE (Genoa, Italy) and used for measuring the contact angle value [31]. During the whole experiment, the total pressure inside the experimental chamber; the temperature on the test table; the heater temperature; and the capillary temperature were simultaneously monitored.

### 2.3. Product Characterization

After the wetting test, the cross-sections of the samples were examined by Field Emission Scanning Electron Microscopy (Zeiss Ultra FESEM, Carl Zeiss AG, Oberkochen, Germany) equipped with an XFlash^®^ 4010 Detector supplied by Bruker Corporation (Billerica, MA, USA) for Energy-Dispersive X-ray Spectroscopy (EDS).

## 3. Results and Discussion

### 3.1. Wetting Behavior

**Figure 2:**
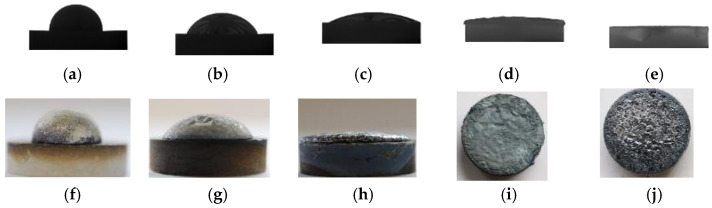
The recorded images and sample images after the wetting test at various temperatures; 973 K (**a**,**f**), 1073 K (**b**,**g**), 1123 K (**c**,**h**), 1223K (**d**,**i**), and 1273 K (**e**,**j**).

**Figure 3:**
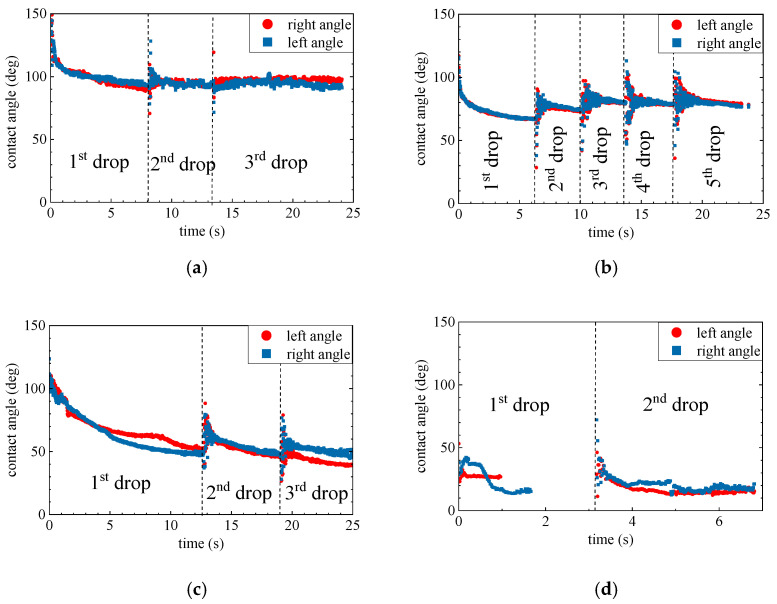
Variation of the left and right contact angles immediately after the deposition of Mg drops at 973 K (**a**), 1073 K (**b**), 1123 K (**c**), and 1173 K (**d**).

**Figure 4:**
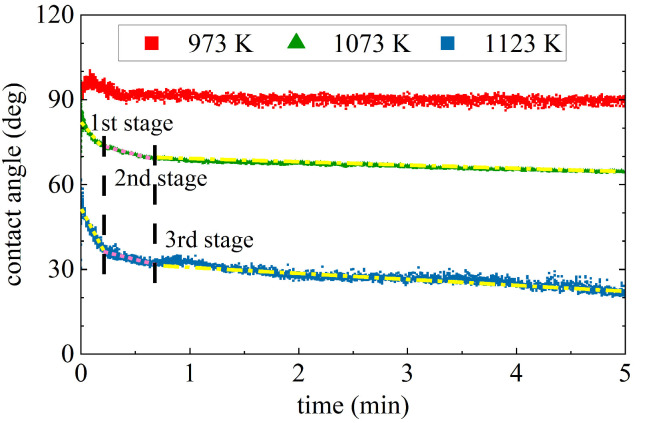
Variation of the average contact angles over time at 973 K, 1073 K, and 1123 K.

The recorded images and sample images after the wetting tests at various temperatures are presented in Figure 2. Recorded videos during the wetting tests are given in the Supplementary Materials, illustrating the change in the shape of the Mg drop during the wetting experiment. In each experiment, three to five drops were deposited on the SiO_2_ substrate to obtain a large enough Mg drop to measure the contact angle of the wetting system. Time zero was set as the first Mg drop was deposited on the substrate. The change in the left and right contact angles after the deposition of each drop with time is shown in Figure 3 at the beginning of the wetting test. At 973 K and 1073 K, the first Mg drop spread on the substrate with an initial decrease in the contact angle, while the contact angle did not change significantly by the deposition of more Mg melt drops, as seen in Figure 3a and Figure 3b, respectively. At 1123 and 1173 K, the Mg drops spread more rapidly on the substrate compared to the lower temperatures, as seen in Figure 3c,d, resulting in very low contact angles. Figure 4 compares the average contact angles at 973, 1073, and 1123 K over a wetting time of 5 min. The calculated average contact angles are the means of right and left contact angles, given in the Supplementary Materials Figure S1. It was observed that the contact angle decreased for the initial 1.5 min of wetting time at 973 K. After the initial period, the contact angle remained almost constant around an average of 90° over time. By increasing temperature to 1073 K, the contact angle decreased slowly with time. It reached an average value of 65° in the period of 4 to 5 min of wetting time. The contact angle also decreased continuously with time at 1123 K, where it was approximately 22° at 5 min of wetting time. At these three temperatures, the decreasing contact angles were accompanied by an increasing drop diameter. At 1073 and 1123 K, the spreading rate can be divided into three stages. In the first stage, the Mg drop spreads rapidly, followed by a slower spreading rate in the second stage and an almost constant contact angle in the third stage. At higher temperatures (1173, 1223, and 1273 K), the rapidly spreading Mg melt drop on the substrate made it impossible to measure contact angles over time. The Mg melt spread over the surface of the substrate and would subsequently either penetrate the substrate or evaporate due to its high vapor pressure. The recorded images by CCD visualize that the Mg melt drops were vibrating during wetting at higher temperatures, indicating Mg evaporation, which can be seen in the videos in the Supplementary Materials. Mg vapor pressures at the different reaction temperatures listed in Table 1 indicate a significant increase in Mg evaporation at 1173 K and above. It is worth noting that at lower temperatures, the Mg drop vibration was not dominant during wetting.

### 3.2. Microstructure Evaluation of the SiO_2_/Mg Interface

**Figure 5:**
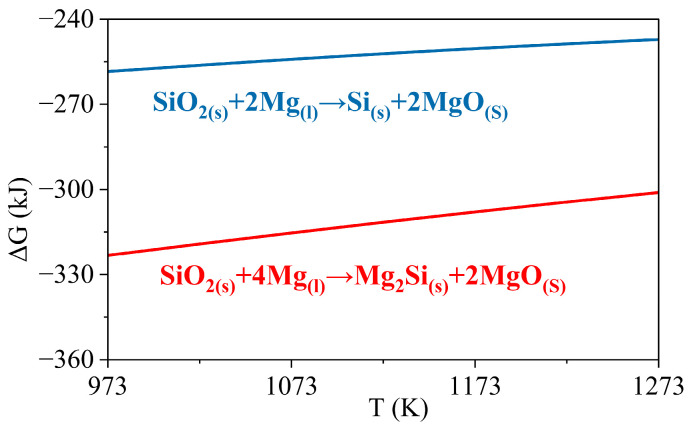
The Gibbs energy of the reaction between SiO_2_ and Mg to form Si-MgO and Mg_2_Si-MgO depends on the Mg available during the reduction reaction, calculated by FactSage 8.1 [32].

SiO_2_-Mg wetting will depend on Mg availability during the reduction reaction, forming Si-MgO and Mg_2_Si-MgO, as shown in Figure 5.

**Figure 6:**
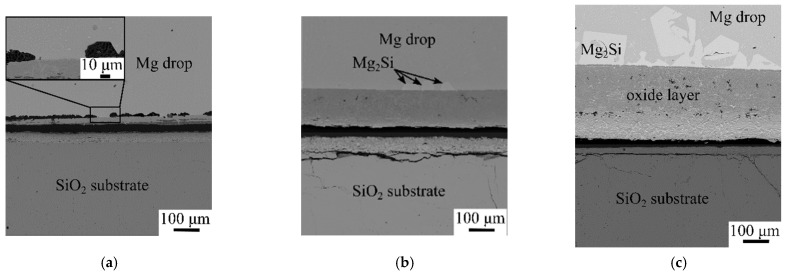
Backscattered electron images of Mg drops and substrates at 973 K (**a**), 1073 K (**b**), and 1123 K (**c**).

**Figure 7:**
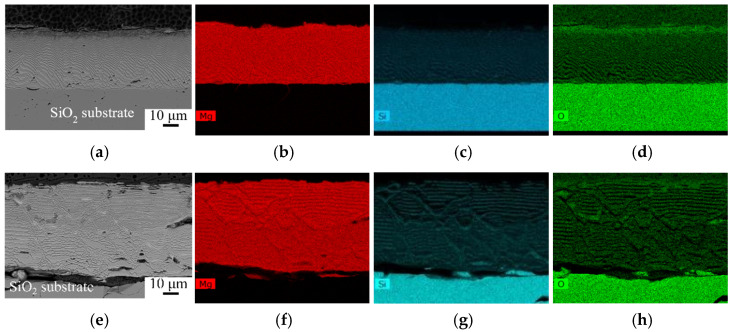
Backscattered electron images and elemental mapping of substrates at 973 K (**a**–**d**) and 1073 K (**e**–**h**).

After the wetting test, the solidified Mg drops were found unattached to the substrates at 973, 1073, and 1123 K. However, product layers were observed on both the Mg drop and substrate surfaces, where the product thickness increased with temperature, as shown in Figure 6. On the Mg drop side, small eutectic Mg_2_Si particles were seen in the Mg matrix at 973 K. Relatively large primary Mg_2_Si crystals, in addition to small particles, were present at 1073 and 1123 K, indicating more Si formation at the higher temperature. The product layer between the Mg drop and the substrate can be divided into two layers. The layer next to the Mg drops mostly contains MgO, as most of the produced Si diffused into the Mg drop. The layer next to the substrate was characterized by a periodic, layered structure composed of Mg_2_Si and MgO-Si layers, as shown in Figure 7. The formation of the periodic layered structure has previously been observed in the SiO_2_/Mg system at a temperature range of 723–1073 K (450–800 °C) where solid-state reactions may take place [12,33,34,35,36]. There are two conditions that should be satisfied to establish the periodic layered structure in the ternary diffusion system. The first condition is that the mobility of one atom should be higher than the other two elements; here, Mg has high mobility compared to Si and O. The second condition is that only one of the product phases can have a thermodynamically stable interface with SiO_2_. Between the Mg_2_Si and MgO phases, the latter was found adjacent to SiO_2_, while the former is unable to establish an interface with SiO_2_ due to the strong tendency of Mg to reduce SiO_2_ and form MgO. There is a strong driving force for Si diffusion out of the MgO phase due to its low wetting; however, MgO is highly resistant to Si diffusion at 923–973 K (650–700 °C) [15,37]. The exact chemical compositions of the layers depend on the degree of phase separation. As seen in Figure 7e, MgO has more porosity, visualized in a slightly darker color in the upper part, indicating a higher degree of Si separation. The lighter gray color of MgO in the lower part implies lower Si separation.

**Figure 8:**
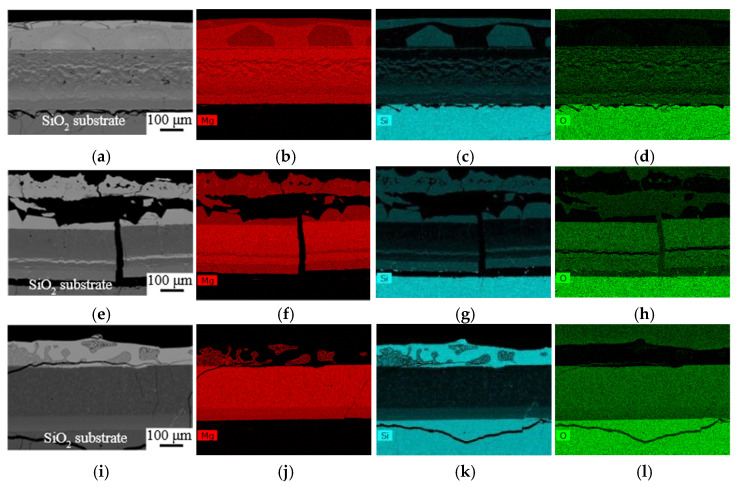
Backscattered electron images and elemental mapping at 1173 K (**a**–**d**), 1223 K (**e**–**h**), and 1273 K (**i**–**l**).

As shown in Figure 8, wetting samples present different microstructures at 1173, 1223, and 1273 K. Three regions are distinguished in the microstructures, including a metal layer on top of two oxide layers with different microstructures. At 1173 K, the metal layer is composed of Mg_2_Si on top and Mg beneath, containing large crystals of Mg_2_Si. The metal layer is Mg_2_Si, containing a small amount of Mg with a large crack at 1223 K. At 1273 K, Si was found on the metal layer with some amount of Mg_2_Si, indicating a higher Mg diffusion rate or evaporation rate at higher temperatures. The intermediate layers at 1173 K and 1223 K contain a relatively coarse Mg_2_Si phase in a MgO matrix, while mainly MgO is found at 1273 K. The layer adjacent to the SiO_2_ substrate is newly formed product phases with no phase separation. Large cracks at the SiO_2_ substrate may originate from released stress due to the volume expansion during the conversion of reactants and during cooling due to differences in the thermal expansion of different phases. The formation of various layers was discussed in detail in our previous works [23,24].

### 3.3. Wetting Mechanism

**Figure 9:**
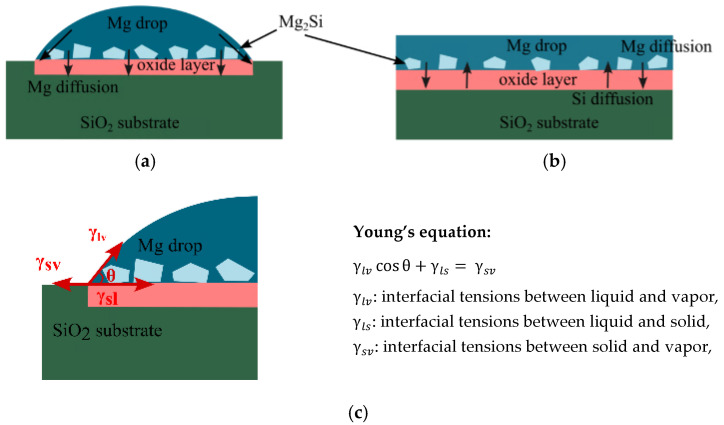
Schematic view of the SiO_2_/Mg wetting test at lower temperatures (<1173 K) (**a**), complete wettability at higher temperatures (>1173 K) (**b**), and (**c**) Young’s equation [41].

In the reactive wetting system, the contact angle and spreading rate of a drop depend on the interfacial reaction [18,38]. With the highly negative Gibbs energy of reduction of SiO_2_ by Mg, as shown in Figure 5, the contribution from the chemical reaction can significantly improve wettability, as reported by Shi et al. [12]. However, the transport rate of reacting species to or from the reaction interface can also impact the interfacial reaction. Here, Mg diffusion through the product layer/SiO_2_ material is the rate-limiting step [10,33,38]. As illustrated in the schematic view in Figure 9, a product layer containing an oxide phase and some Mg_2_Si particles formed between the Mg drop and SiO_2_. The diffusion rate of Mg through these product layers to the triple junction, where the gas phase, Mg liquid, and SiO_2_ substrate meet, governs the contact angle and spreading rate of the Mg drop. Moreover, the interfacial tension between various formed phases affects the wettability of SiO_2_ by Mg and makes the wetting system quite complex, as shown in Figure 9c.

**Figure 10:**
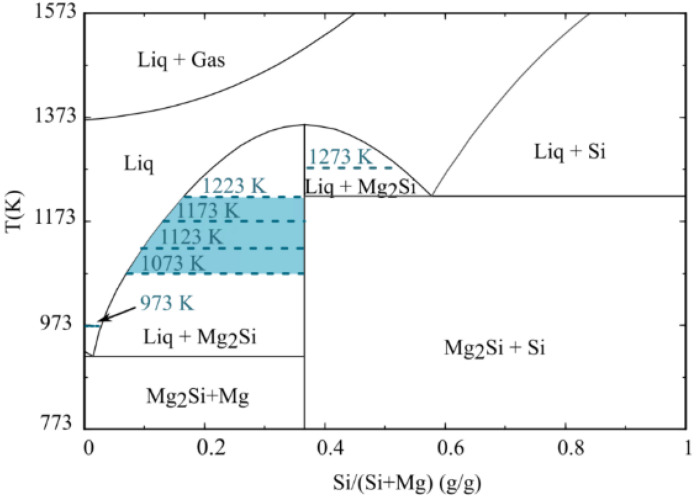
The binary phase diagram of Mg-Si, plotted by FactSage 8.1 [32].

Furthermore, Mg has a surface tension of 559 mN.m^−1^ at its melting point, 928 K, whereas the surface tension of Si at its melting point, 1683 K, is 865 mN.m^−1^ [39]. The produced Si dissolved in the Mg drop leads to increasing surface tension of the drop at a constant temperature, consequently slowing down the spreading rate. As seen in Figure 7 and Figure 8, the top metal layer was composed of different phases, including Mg, Mg_2_Si, and Si at different temperatures. The binary phase diagram of Mg-Si in Figure 10 illustrates the produced Si initially dissolved in the Mg melt. Only eutectic Mg_2_Si formed at 973 K, while the formation of primary Mg_2_Si is observed at higher temperatures in the form of small particles. At temperatures up to 1223 K, all produced Si was found in the Mg_2_Si phase, indicating that the metal phase had a chemical composition on the left side of the Mg_2_Si line in the binary phase diagram. At 1273 K, the presence of Si and Mg_2_Si phases implies higher Si formation and higher Mg loss by evaporation, which introduced a metal phase with a chemical composition on the right side of the Mg_2_Si line, containing Mg_2_Si and Si. Moreover, Si reduces the viscosity of the Mg melt but also its spreading rate [40]. On the other hand, with increasing temperature, both the surface tension and viscosity of the Mg drop decrease, which together with a higher Mg diffusion rate, improve wettability [39].

## 4. Conclusions

An investigation of the wetting behavior of Mg on SiO_2_ substrates showed a reactive wetting system, where the wettability increased with the formation of product phases. The measured contact angles after 5 min of wetting time were 90°, 65°, and 22°, respectively at 973, 1073, and 1123 K. By increasing the temperature to 1173 K and above, wettability improved significantly with the Mg melt drop spreading rapidly on the SiO_2_ substrate. The higher contact angles were accompanied by a less developed interfacial area between SiO_2_ and Mg at lower temperatures, i.e., the wetting behavior of the SiO_2_/Mg system influenced the reaction kinetics through the extent of the interfacial area between the reactants. It can be concluded that reaction kinetics are dependent on temperature, affecting both the wetting behavior between SiO_2_ and Mg and the diffusion rate of Mg. However, the influence of the Mg diffusion rate appeared to be more pronounced, particularly at higher temperatures.

Furthermore, various microstructures were found at different wetting temperatures. During the wetting test, there were two fluxes of atoms in opposite directions: Mg diffused in a long range towards the reaction zone while Si diffused towards the Mg drop with a diffusion rate depending on temperature. At lower temperatures, Si could only diffuse a short distance, which led to its accumulation as thin layers between the MgO layers and the formation of the periodic layered structure. In contrast, at higher temperatures, Si diffused out through the MgO oxide layer to accumulate in the Mg melt on top. It can be stated that 1173 K represents a borderline temperature where a periodic layered reaction product structure was observed below this temperature. The separation of Si from the MgO-Si phase due to their low wettability occurred over the wetting time, where late-formed MgO-Si layers had a higher Si content compared to earlier-formed layers.

## Figures and Tables

**Table 1:** Vapor pressure of Mg at different wetting temperatures, calculated by FactSage 8.1.

Temperature (K)	973	1073	1123	1173	1223	1273
Vapor pressure (atm)	9.4 × 10^−3^	4.3 × 10^−2^	8.4 × 10^−2^	1.5 × 10^−1^	2.6 × 10^−1^	4.32 × 10^−1^

## Data Availability

Data are contained within the article and Supplementary Materials.

## Supplementary Materials

The following supporting information can be downloaded at https://www.mdpi.com/article/10.3390/ma17061302/s1, Figure S1: Variation of right and left contact angles with wetting time at 973 K, 1073 K, and 1123 K.; Figure S2. EDX point analysis at 973 K. Figure S3. EDX point analysis at 1073 K. Figure S4. EDX point analysis at 1123 K. Figure S5. EDX point analysis at 1173 K. videos of the wetting experiment.
