# Supplementary material for: High-Temperature Reactive Wetting of Natural Quartz by Liquid Magnesium
Source: Materials (Basel). 2024 Mar 11;17(6):1302. doi: 10.3390/ma17061302 (PMC10972395; doi:10.3390/ma17061302)
Supplement: Supplementary file 1 [file materials-17-01302-s001.zip › materials-2809360-supplementary/materials-2809360-supplementary.pdf]

materials

MDPI

Supplementary Materials

High-Temperature Reactive Wetting of Natural Quartz by Liquid Magnesium

Azam Rasouli [1,*], Artur Kudyba [2], Grzegorz Bruzda [2], Jafar Safarian [1], and Gabriella Tranell [1]

[1] Department of Materials Science and Engineering, Norwegian University of Science and Technology, Trondheim, Norway
[2] Łukasiewicz Research Network – Kraków Institute of Technology, Kraków, Poland
* Correspondence: azam.rasouli@ntnu.no

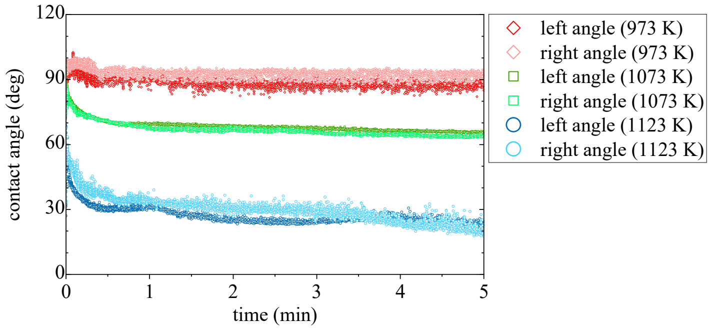

Figure S1. Variation of right and left contact angles with wetting time at 973 K, 1073 K, and 1123 K.

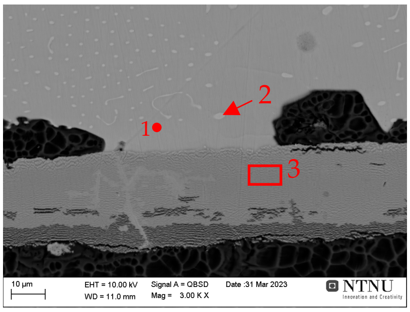

point	Element concentration (at%)		
	Si	Mg	O
1	-	100.00	-
2	36.62	63.11	0.26
3	3.6	79.48	16.92

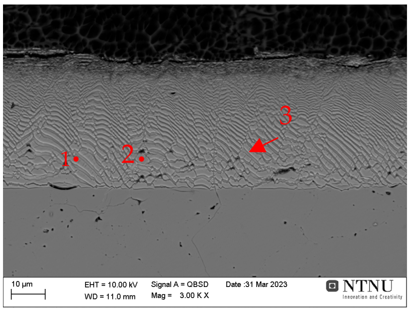

poin t	Element noncentration (at%)		
	Si	Mg	O
1	13.80	61.59	24.61
2	15.62	61.43	22.95
3	34.67	59.65	5.67

Figure S2. EDX point analysis at 973 K.

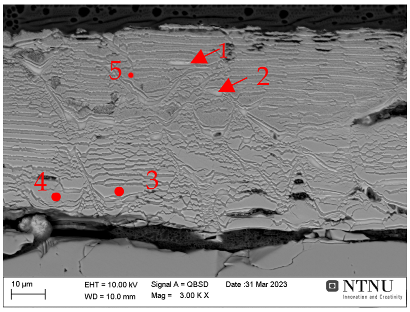

point	Element concentration (at%)		
	Si	Mg	O
1	38.70	61.30	-
2	37.55	59.45	3.00
3	15.16	62.30	22.54
4	18.224	61.87	19.89
5	4.58	71.12	23.30

Figure S3. EDX point analysis at 1073 K.

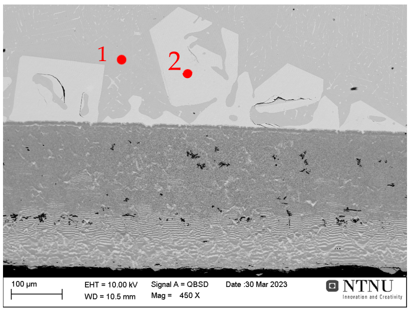

point	Element concentration (at%)		
	Si	Mg	O
1	-	99.97	0.03
2	38.28	61.72	-

Figure S4. EDX point analysis at 1123 K.

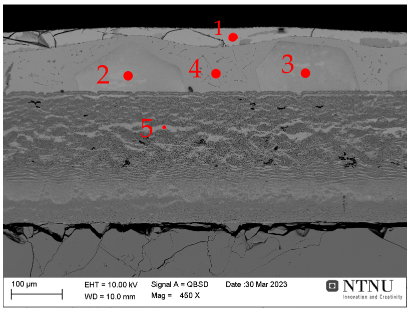

point	Element concentration (at%)		
	Si	Mg	O
1	38.63	61.37	-
2	38.95	59.82	1.23
3	39.58	60.42	-
4	-	100.00	-
5	38.96	58.29	2.75

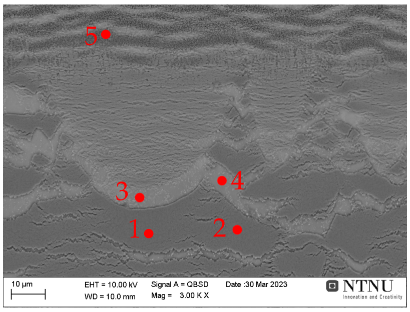

point	Element concentration (at%)		
	Si	Mg	O
1	14.73	62.45	22.82
2	15.08	62.23	22.68
3	38.41	57.62	3.97
4	37.78	55.87	6.35
5	4.58	70.18	25.24

Figure S5. EDX point analysis at 1173 K.
